# Electroconvulsive therapy practice during the COVID-19 pandemic

**DOI:** 10.6061/clinics/2020/e2056

**Published:** 2020-06-16

**Authors:** Helena Bellini, Eric Cretaz, Luiz Felipe Rigonatti, Carla Dominique Rodrigues De Conto, Débora Luciana Melzer-Ribeiro, Geraldo Busatto-Filho, André Russowsky Brunoni, José Gallucci-Neto

**Affiliations:** I Instituto de Psiquiatria, Hospital das Clinicas HCFMUSP, Faculdade de Medicina, Universidade de Sao Paulo, Sao Paulo, SP, BR.

Between December 31, 2019, and January 3, 2020, a total of 44 patients presented in Wuhan, Hubei Province, China, with pneumonia of unknown etiology. By January 7, 2020, scientists had isolated a new type of coronavirus, severe acute respiratory syndrome coronavirus 2 (SARS-CoV-2), from these patients. It was hypothesized that the outbreak was associated with a seafood market in Wuhan (1).

China promptly adopted public health measures to prevent and control the epidemic, including isolating patients, monitoring their contacts, and developing diagnostic and treatment procedures (2). However, the number of confirmed cases continued to increase, and the disease spread to other countries.

The first cases of coronavirus disease (COVID-19) outside China were reported in Thailand, Japan, and Korea. Since then, COVID-19 has spread to American and European territories. By the time the World Health Organization declared a pandemic on March 11, 2020, there were approximately 118,000 cases of COVID-19, with 4,291 deaths in 114 countries (1).

In the American continent, the first case was reported in the United States of America on January 21, 2020, followed by Canada, which reported its first case four days later. In South America, the first case was confirmed on February 26, 2020 in the city of São Paulo, Brazil. By May 7, 2020, the country had already 135,106 confirmed cases and 9,146 deaths (3). However, these numbers are likely underreported. In addition to the absence of proper screening mechanisms for asymptomatic patients (4), Brazil's health system suffers from a chronic lack of testing for suspected cases and deaths, as is the case in a number of developing countries.

## Clinical characteristics of COVID-19

SARS-CoV-2 may remain viable in the environment for up to 72 hours (5). Besides, an infected respiratory droplet can spread viral loads in the environment up to 2 meters from where it is generated through coughing, sneezing, or speaking. In this manner, COVID-19 is acquired either by inhaling droplets or touching a contaminated surface and then touching the nose, mouth, or eyes. An infected patient usually transmits the disease for an average period of 7 days after the onset of symptoms. However, preliminary data suggests that transmission may occur even without the appearance of any symptoms (6).

According to a recent meta-analysis, the most common clinical symptoms of COVID-19 are fever, cough, myalgia, fatigue, expectoration, and dyspnea (7). Besides, headache, dizziness, sore throat, and gastrointestinal symptoms have also been described (8). The intensity of symptoms suffered may vary. While the majority of individuals are asymptomatic, clinical presentations range from mild symptoms to pneumonia and can lead to death (9). The most severe complications include acute respiratory distress syndrome, arrhythmia, and shock (10), with intensive care admission rates of approximately 25 to 30% (9). Depending on the patient's clinical profile, the mortality rate associated with COVID-19 is estimated to range from 2 to 15% (7). There are no known treatments or vaccines.

While everyone is susceptible to COVID-19, some patients may have a higher risk of developing the most severe forms of the disease. Such cases are associated with a higher rate of hospitalization, worse prognosis, and higher mortality rate (10,11). People more at risk include the elderly, smokers, and those with underlying comorbidities, such as cardiovascular and cerebrovascular disease, hypertension, diabetes mellitus, chronic obstructive pulmonary disease, malignancy, and chronic kidney disease (12,13).

## Vulnerability of patients with a psychiatric condition

It is well-known that a pandemic can result in a range of psychiatric morbidities in the general population (14). Similarly, considerable widespread psychological impact has been reported after a quarantine period (15). Wang et al. (16) reported that more than half of 1,210 random subjects from 194 Chinese cities rated their psychological impact during the initial stage of the COVID-19 outbreak as moderate to severe. Despite the lack of research, it is reasonable to hypothesize that people with a history of mental illness may be more sensitive to the psychological effects of a pandemic, increasing the risk of a mental relapse (17).

In addition to the worsening of their psychopathological symptoms, psychiatric patients have a higher risk of contracting an infectious disease than does the general population, including pneumonia (18). In this regard, inpatients seem especially vulnerable. During the initial periods of the pandemic, a Chinese newspaper reported that at least 50 patients and 30 mental health professionals in Wuhan's largest psychiatric hospital had COVID-19. The intense interpersonal contact inherent to a psychiatric unit promotes the right conditions for the rapid dissemination of any etiological agent. Inpatients frequently exhibit behavioral disorganization or cognitive impairments that compromise their ability to understand risks and adhere to preventive and hygiene measures. In addition, mental health professionals themselves are often not routinely trained to deal with infectious diseases (19).

Patients with mental health conditions that have COVID-19 have higher hospitalization rates and worse prognosis, especially those with more severe disorders, in whom there is a high prevalence of medical comorbidities in this population (20). According to a recent review (21), patients with schizophrenia or bipolar affective disorder have an increased risk of cardiovascular (*e.g.*, coronary heart disease, heart failure, hypertension, angina, myocardial infarction), cerebrovascular (*e.g.*, stroke, transient ischemic attacks), endocrine (*e.g.*, diabetes mellitus, thyroid disorders), respiratory (*e.g.*, asthma, chronic obstructive pulmonary disease), infectious (*e.g.*, hepatitis C, immune deficiency and autoimmune diseases. These effects appear to be secondary to both mental illness and the use of psychotropic drugs. Depression has also been associated with cardiovascular disease, immunosuppression, and cancer (22)**.** Finally, higher rates of smoking in patients with psychiatric disorders have been well-described (23).

## The COVID-19 pandemic and electroconvulsive therapy services

Electroconvulsive therapy (ECT) is considered one of the most effective and safe interventions in psychiatry, particularly for cases that require a rapid therapeutic response, such as those associated with suicidal ideation, progressive clinical deterioration, or catatonia (24). Such characteristics have several implications in the context of the COVID-19 pandemic. First, ECT services often bring together elderly (25) and critically ill individuals, who have higher rates of medical comorbidities (20), poorer hygiene (26), and worse cognitive profiles (27). This population has a higher COVID-19 mortality rate (7).

Besides, during a pandemic, health resources are scarce and non-vital services may be closed or repurposed. Despite its importance in life-threatening situations, ECT has historically been classified as an elective treatment, so many ECT services may be limited. This can have catastrophic consequences for patients with severe mental health disorders (28), especially considering that such a social context can trigger and aggravate psychiatric symptoms (14-16). Unfortunately, a team from a general hospital in Singapore (29) has already reported the suicide of a patient with depression whose ECT treatment was impaired by limited anesthetic resources because of the COVID-19 pandemic.

Another implication for ECT units is related to anesthetic practices required for the procedure. ECT is universally performed under general anesthesia, to provide comfort and safety. After anesthetic induction, the patient's airways are usually maintained with bag-mask ventilation, whereas endotracheal intubation is rarely required (30). Such interventions, in particular non-invasive ventilation, may generate a localized aerosol, which carries a high risk of contagion to those closely involved in the procedure (31). Thus, there is a risk for both patients and staff.

As such, there are many challenges with the delivery of ECT in the current COVID-19 pandemic. Efficacious strategies need to be identified so that ECT services can continue to operate throughout the COVID-19 pandemic, according to available resources.

## ECT service at the University of São Paulo medical school

The mental health system is precarious in Brazil, especially regarding ECT. The population of Brazil exceeds 210 million, of which 10 million live in the city of São Paulo, one of the most populated metro areas worldwide (32). To the best of our knowledge, there are only 11 institutions that offer ECT free of charge to patients across the country, meaning that there is one public ECT service for every 19 million inhabitants. Most large cities do not have this kind of service because the procedure is not financed by the Ministry of Health. There are private clinics for those that can afford to pay; however, the only option for those that cannot are public institutions linked to medical universities. Nevertheless, both are hard to find (33).

The Institute of Psychiatry of Clinics Hospital of the University of São Paulo Medical School (IPq-HCFMUSP) offers outpatient and hospital treatments for a range of mental health disorders, including neuromodulation techniques and other biological therapies. The Institute assists in research, teaching, and assistance for all of South America, integrating a hospital complex (HCFMUSP) that offers 2,400 hospital beds and 600,000 outpatient consultations to the public each year.

Our ECT service performs between 500 and 600 ECT procedures monthly. Of these, 75% are delivered to outpatients and 25% to inpatients. The opening hours are from 8 a.m. to 3 p.m. Because of the lack of ECT units in the country, the service treats patients from several different geographic regions and social classes. Regarding finance, most assistance is provided through the Institute's limited resources.

The risk of contamination within the ECT service of IPq-HCFMUSP during the COVID-19 pandemic is especially high because of several factors. First, it is an outpatient clinic, so there is the potential for an infected person to present. Moreover, ECT patients often have difficulties in understanding COVID-19 prevention measures. Second, as the service assists both outpatients and inpatients, the virus may enter the wards, contaminating other patients who are not receiving ECT. Third, patient turnover is remarkably high, and there is close and repetitive contact between patients, their peers, and health professionals, inside a confined environment with restricted air circulation.

In addition to contamination, lack of assistance is also a risk factor. Most patients travel long distances to arrive at the Institute. Because of the isolation measures in place to control the pandemic, mobility within and outside the city is restricted, preventing some patients from accessing treatment. Moreover, being a part of a hospital complex, the facilities and resources of the ECT service may be repurposed to treat patients with COVID-19, which would drastically decrease its regular functioning. Considering its importance and the lack of other similar units, the abrupt closure of our ECT service would severely impact the delivery of ECT across much of the country.

## Measures to deal with the COVID-19 pandemic and its immediate impacts

Since the quarantine was decreed by the Governor of the State of São Paulo on February 24, 2020 (34), all people with COVID-19 were isolated at the Central Institute of HCFMUSP, whereas other cases were transferred to the remaining units. For instance, IPq - HCFMUSP received patients from Neurology and Neurosurgery departments.

We also made some changes to the ECT service facilities to reduce the risk of contamination, according to the National Health Surveillance Agency's guidelines (35). From February 24, 2020, all individuals who require psychiatric hospitalization are first being admitted to an isolated ward for 14 days before being transferred to a regular ward. In addition, visits by family members are limited. As a result of these measures, we considered that the risk of contamination was lower for inpatients than for outpatients, and we divided the service period into two shifts, the first one reserved exclusively for inpatients and the second, for outpatients.

For both groups of patients, we are providing educational pamphlets (guidelines on hygiene and respiratory etiquette) and hand sanitizer. In particular, for outpatients, we have increased the frequency of cleaning of the waiting room, and the chairs have been spread further apart with the windows left open to improve ventilation. Waiting time has also been drastically reduced. Additionally, we are screening both outpatients and their families (now limited to one per patient) for respiratory symptoms on arrival. In the case of suspected COVID-19, appropriate preventive actions are taken, and the patient is immediately referred to the emergency department.

Regarding environmental measures, internal facilities with frequent personal contact are cleaned with 70% alcohol after each patient leaves to reduce the risk of contamination between patients. The internal areas undergo daily terminal cleaning with a neutral detergent. Afterward, the surfaces are disinfected with chlorine-based products, especially the most frequently touched surfaces and equipment (*e.g.*, door handles, telephones, tables, light switches, handrails and grab bars, handles, gauntlets, and monitoring equipment). The entire process is carefully supervised by our nurse manager, who also ensures proper use of personal protective equipment (PPE) by the entire team.

The PPE components were selected on the basis of the type of contact each professional has with patients. In addition to thoroughly cleaning their hands, support professionals who work at reception or security have been instructed to wear a surgical mask, gown, and procedure gloves. They continued, as usual, to welcome patients through a glass panel. Doctors and nurses have been instructed to wear full PPE (N95 masks, face shield, fluid-resistant gown, gloves, and hoods) because ECT is considered an aerosol-generating procedure. We reduced the number of practitioners in the ECT application room to the minimum necessary, and all staff have received training on how to use PPE correctly. In addition, staff undergo periodic screening for respiratory symptoms. At the time of this report, there were two reported cases of infection in our team, one receptionist and one anesthesiologist. Both of them remained isolated for 10 days and have fully recovered.

Regarding the ECT procedure, the most important change made was in relation to anesthesia. According to our previous protocol, we kept using disposable bite blocks and exhalation filters attached to the bag-mask. Typically, anesthetic induction includes etomidate 0.15-0.2 mg/kg IV or propofol 1.0 mg/kg IV and succinylcholine 0.5-0.6 mg/kg IV, followed by bag-mask ventilation with 100% oxygen. We have decreased the dose of succinylcholine to 0.3-0.4 mg/kg IV and replaced the bag-mask ventilation with 100% preoxygenation for 5 minutes. Interestingly, the side effects and complication rates have remained the same, whereas anesthetic recovery time is shorter for some patients. Indeed, the results of Nazemroaya et al. (36) suggest that preoxygenation with 100% oxygen for 5 minutes is comparable to bag-mask ventilation for ECT in terms of O2 saturation.

**Figure 1:**
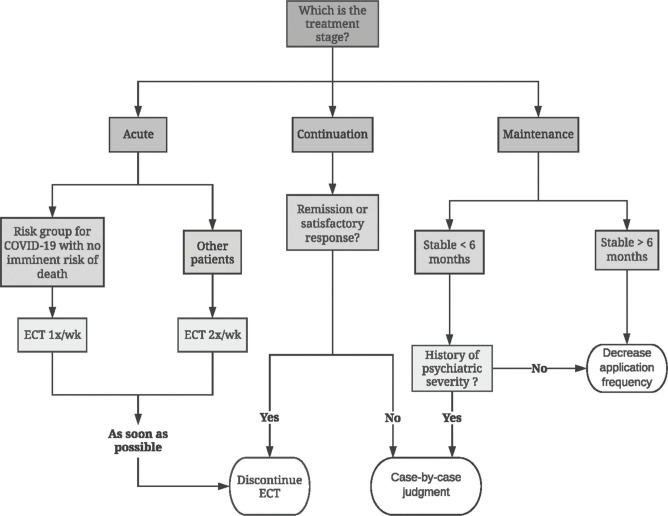
Instructions for reassessing electroconvulsive therapy (ECT) frequency.

Finally, our team has been evaluating the possibility of reducing the frequency of sessions for each patient. In this, we consider factors such as the treatment stage and comorbidities (Figure 1), in addition to the opinion of the referring psychiatrist. Once a patient's treatment regimen has been changed, he or she is routinely screened to prevent mental health problems from worsening.

**Figure 2:**
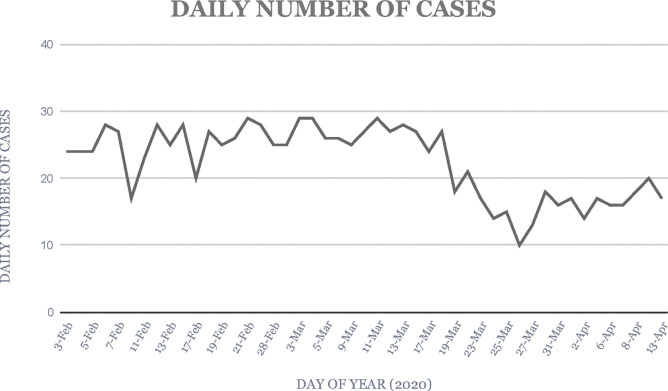
Daily electroconvulsive therapy (ECT) procedures before and after the pandemic.

**Figure 3:**
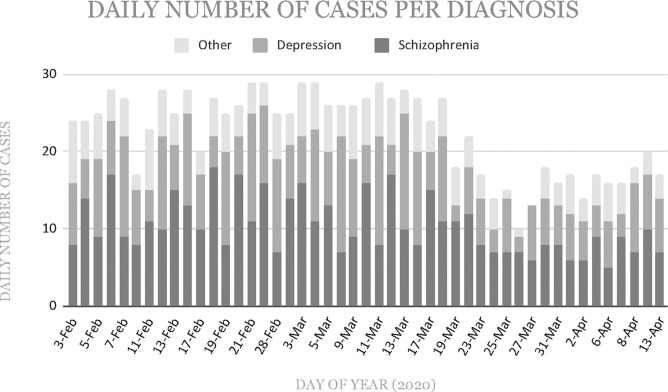
Daily electroconvulsive therapy (ECT) procedures per diagnosis.

Right after the pandemic, there was a decrease in the daily number of procedures (Figure 2 and Figure 3). Until May 7, 2020, two of our patients have died from COVID-19, a young male and a female, both on maintenance ECT for schizophrenia. Another patient, who had been receiving ECT for a severe depressive episode, failed to attend treatment because of reduced mobility in the city. Although a causal relationship cannot be established, he had an unprecedented seizure right after the abrupt stop of ECT.

## Final considerations

The COVID-19 pandemic has been unprecedented. Some experts believe that COVID-19 control strategies may be required for at least another 2 years or more (37), which could severely impact ECT services. Considering the indispensable role of ECT, our report aims to promote communication and to draw attention to ECT units during the COVID-19 pandemic, emphasizing the importance of developing strategies to mitigate its impact on ECT practice.

## References

[1] Coronavirus (2020). https://www.who.int/health-topics/coronavirus#tab=tab_1.

[2] Wang C, Horby PW, Hayden FG, Gao GF (2020). A novel coronavirus outbreak of global health concern. Lancet.

[3] Health — Brazil (2020). http://www.brazil.gov.br/government/ministers/health.

[4] Wilder-Smith A, Chiew CJ, Lee VJ (2020). Can we contain the COVID-19 outbreak with the same measures as for SARS?. Lancet Infect Dis.

[5] van Doremalen N, Bushmaker T, Morris DH, Holbrook MG, Gamble A, Williamson BN (2020). Aerosol and Surface Stability of SARS-CoV-2 as Compared with SARS-CoV-1. N Engl J Med.

[6] Rabi FA, Al Zoubi MS, Kasasbeh GA, Salameh DM, Al-Nasser AD (2020). SARS-CoV-2 and Coronavirus Disease 2019: What We Know So Far. Pathogens.

[7] Li LQ, Huang T, Wang YQ, Wang ZP, Liang Y, Huang TB (2020). COVID-19 patients' clinical characteristics, discharge rate and fatality rate of meta‐analysis. J Med Virol.

[8] Chen N, Zhou M, Dong X, Qu J, Gong F, Han Y (2020). Epidemiological and clinical characteristics of 99 cases of 2019 novel coronavirus pneumonia in Wuhan, China: a descriptive study. Lancet.

[9] Singhal T (2020). A Review of Coronavirus Disease-2019 (COVID-19). Indian J Pediatr.

[10] Wang D, Hu B, Hu C, Zhu F, Liu X, Zhang J (2020). Clinical Characteristics of 138 Hospitalized Patients With 2019 Novel Coronavirus-Infected Pneumonia in Wuhan, China. JAMA.

[11] Liu K, Chen Y, Lin R, Han K (2020). Clinical features of COVID-19 in elderly patients: A comparison with young and middle-aged patients. J Infect.

[12] Emami A, Javanmardi F, Pirbonyeh N, Akbari A (2020). Prevalence of Underlying Diseases in Hospitalized Patients with COVID-19: a Systematic Review and Meta-Analysis. Arch Acad Emerg Med.

[13] Li B, Yang J, Zhao F, Zhi L, Wang X, Liu L (2020). Prevalence and impact of cardiovascular metabolic diseases on COVID-19 in China. Clin Res Cardiol.

[14] Yi Y, Lagniton PNP, Ye S, Li E, Xu RH (2020). COVID-19: what has been learned and to be learned about the novel coronavirus disease. Int J Biol Sci.

[15] Brooks SK, Webster RK, Smith LE, Woodland L, Wessely S, Greenberg N (2020). The psychological impact of quarantine and how to reduce it: rapid review of the evidence. Lancet.

[16] Wang C, Pan R, Wan X, Tan Y, Xu L, Ho CS (2020). Immediate Psychological Responses and Associated Factors during the Initial Stage of the 2019 Coronavirus Disease (COVID-19) Epidemic among the General Population in China. Int J Environ Res Public Health.

[17] Yao H, Chen JH, Xu YF (2020). Patients with mental health disorders in the COVID-19 epidemic. Lancet Psychiatry.

[18] Seminog OO, Goldacre MJ (2013). Risk of pneumonia and pneumococcal disease in people with severe mental illness: English record linkage studies. Thorax.

[19] Xiang YT, Zhao YJ, Liu ZH, Li XH, Zhao N, Cheung T (2020). The COVID-19 outbreak and psychiatric hospitals in China: managing challenges through mental health service reform. Int J Biol Sci.

[20] Casey DE (2005). Metabolic issues and cardiovascular disease in patients with psychiatric disorders. Am J Med.

[21] Sinha A, Shariq A, Said K, Sharma A, Jeffrey Newport D, Salloum IM (2018). Medical Comorbidities in Bipolar Disorder. Curr Psychiatry Rep.

[22] Benton T, Staab J, Evans DL (2007). Medical co-morbidity in depressive disorders. Ann Clin Psychiatry.

[23] Kalman D, Morissette SB, George TP (2005). Co-morbidity of smoking in patients with psychiatric and substance use disorders. Am J Addict.

[24] Weiner RD, Reti IM (2017). Key updates in the clinical application of electroconvulsive therapy. Int Rev Psychiatry.

[25] Slade EP, Jahn DR, Regenold WT, Case BG (2017). Association of Electroconvulsive Therapy With Psychiatric Readmissions in US Hospitals. JAMA Psychiatry.

[26] Brewer WJ, Edwards J, Anderson V, Robinson T, Pantelis C (1996). Neuropsychological, olfactory, and hygiene deficits in men with negative symptom schizophrenia. Biol Psychiatry.

[27] Harvey PD, Rosenthal JB (2018). Cognitive and functional deficits in people with schizophrenia: Evidence for accelerated or exaggerated aging?. Schizophr Res.

[28] Espinoza RT, Kellner CH, McCall WV (2020). ECT during COVID-19: An Essential Medical Procedure - Maintaining Service Viability and Accessibility. J ECT.

[29] Tor PC, Phu AHH, Koh DSH, Mok YM (2020). ECT in a time of COVID-19. J ECT.

[30] Chawla N (2020). Anesthesia for Electroconvulsive Therapy. Anesthesiol Clin.

[31] Wax RS, Christian MD (2020). Practical recommendations for critical care and anesthesiology teams caring for novel coronavirus (2019-nCoV) patients. Can J Anaesth.

[32] 2020 World Population by Country (2020). https://worldpopulationreview.com/.

[33] Ribeiro RB, Melzer-Ribeiro DL, Rigonatti SP, Cordeiro Q (2012). Electroconvulsive therapy in Brazil after the “psychiatric reform”: a public health problem-example from a university service. J ECT.

[34] Governo decreta quarentena em todos os municípios do Estado de São Paulo a partir da próxima terça-feira — Prefeitura (2020). http://www.capital.sp.gov.br/noticia/governo-decreta-quarentena-em-todos-os-municipios-do-estado-de-sao-paulo-a-partir-da-proxima-terca-feira.

[35] NOTA TÉCNICA No 04/2020 GVIMS/GGTES/ANVISA (2020). https://www20.anvisa.gov.br/segurancadopaciente/index.php/alertas/item/nota-tecnica-n-04-2020-gvims-ggtes-anvisa?category_id=244.

[36] Nazemroaya B, Shetabi H, Mohammadi S (2018). The effects of different preoxygenation techniques on heart rate and blood pressure alterations in patients undergoing electroconvulsive therapy. J Isfahan Med Sch.

[37] Kissler SM, Tedijanto C, Goldstein E, Grad YH, Lipsitch M (2020). Projecting the transmission dynamics of SARS-CoV-2 through the postpandemic period. Science.

